# PKR deficiency alters *E. coli*-induced sickness behaviors but does not exacerbate neuroimmune responses or bacterial load

**DOI:** 10.1186/s12974-015-0433-2

**Published:** 2015-11-19

**Authors:** David Chun-Hei Poon, Yuen-Shan Ho, Ran You, Hei-Long Tse, Kin Chiu, Raymond Chuen-Chung Chang

**Affiliations:** Laboratory of Neurodegenerative Diseases, School of Biomedical Sciences, LKS Faculty of Medicine, The University of Hong Kong, Hong Kong SAR, China; Research Centre of Heart, Brain, Hormone and Healthy Aging, LKS Faculty of Medicine, The University of Hong Kong, Hong Kong SAR, China; State Key Laboratory of Brain and Cognitive Sciences, The University of Hong Kong, Hong Kong SAR, China; School of Nursing, Faculty of Health and Social Sciences, The Hong Kong Polytechnic University, Hung Hom, Kowloon, Hong Kong SAR, China; Department of Ophthalmology, LKS Faculty of Medicine, The University of Hong Kong, Hong Kong SAR, China; Rm. L1-49, Laboratory Block, Faculty of Medicine Building, 21 Sassoon Road, Pokfulam, Hong Kong SAR, China

**Keywords:** Systemic inflammation, Neuroimmune activation, Neuroinflammation, Sickness behavior, Protein kinase R

## Abstract

**Background:**

Systemic inflammation induces neuroimmune activation, ultimately leading to sickness (e.g., fever, anorexia, motor impairments, exploratory deficits, and social withdrawal). In this study, we evaluated the role of protein kinase R (PKR), a serine-threonine kinase that can control systemic inflammation, on neuroimmune responses and sickness.

**Methods:**

Wild-type (WT) PKR+/+ mice and PKR−/− mice were subcutaneously injected with live *Escherichia coli* (*E. coli*) or vehicle. Food consumption, rotarod test performance, burrowing, open field activity, object investigation, and social interaction were monitored. Plasma TNF-α and corticosterone were measured by ELISA. The percentage of neutrophils in blood was deduced from blood smears. Inflammatory gene expression (IL-1β, TNF-α, IL-6, cyclooxygenase (COX)-2, iNOS) in the liver and the brain (hypothalamus and hippocampus) were quantified by real-time PCR. Blood and lavage fluid (injection site) were collected for microbiological plate count and for real-time PCR of bacterial 16S ribosomal DNA (rDNA). Corticotrophin-releasing hormone (CRH) expression in the hypothalamus was also determined by real-time PCR.

**Results:**

Deficiency of PKR diminished peripheral inflammatory responses following *E. coli* challenge. However, while the core components of sickness (anorexia and motor impairments) were similar between both strains of mice, the behavioral components of sickness (reduced burrowing, exploratory activity deficits, and social withdrawal) were only observable in PKR−/− mice but not in WT mice. Such alteration of behavioral components was unlikely to be caused by exaggerated neuroimmune activation, by an impaired host defense to the infection, or due to a dysregulated corticosterone response, because both strains of mice displayed similar neuroimmune responses, bacterial titers, and plasma corticosterone profiles throughout the course of infection. Nevertheless, the induction of hypothalamic corticotrophin-releasing hormone (CRH) by *E. coli* was delayed in PKR−/− mice relative to WT mice, suggesting that PKR deficiency may postpone the CRH response during systemic inflammation.

**Conclusions:**

Taken together, our findings show that (1) loss of PKR could alter *E. coli*-induced sickness behaviors and (2) this was unlikely to be due to exacerbated neuroimmune activation, (3) elevated bacterial load, or (4) dysregulation in the corticosterone response. Further studies can address the role of PKR in the CRH response together with its consequence on sickness.

## Background

Sickness refers to a set of physiological and behavioral responses (e.g., fever, anorexia, immobility, reduced exploratory activity, social withdrawal, anhedonia) to systemic inflammation [1–3]. Collectively, these changes serve as the body’s adaptive strategies to combat infections and injuries [4]. However, increasing lines of evidence have indicated that the same responses can become deleterious if exacerbated [1, 5–7]. Therefore, it will be beneficial to understand how sickness is regulated.

The regulation of sickness is a complex subject. Upon an inflammatory insult, innate immune cells sense pathogen-associated molecular patterns (PAMPs) and alarmins that are respectively released from pathogens and damaged tissues [8, 9], and respond by upregulating cytokines [10, 11], prostaglandins [12, 13], and complement factors [14, 15]. These systemic inflammatory mediators communicate to the brain via multiple humoral and neural routes, causing neuroimmune activation and sickness [16–20]. Accordingly, inflammation is important for sickness development, and a reduction of inflammation should help to suppress sickness. Indeed, both peripheral [10, 17, 21, 22] and central [23–26] administration of exogenous inflammatory molecules acutely triggers sickness. On the contrary, pharmacological inhibition of the synthesis [27–30] or the actions [31–34] of endogenous inflammatory mediators can decrease sickness following immune challenge by lipopolysaccharide (LPS).

Protein kinase R (PKR) is a ubiquitously expressed serine-threonine kinase that was originally discovered as an antiviral defense mediator [35, 36]. Upon viral infection, PKR binds to double-stranded RNA (dsRNA) from viruses through its N-terminal dsRNA-binding domain, resulting in PKR dimerization and autophosphorylation and activation of the C-terminal kinase domain [37–39]. Activated PKR can inhibit viral infection by phosphorylating eukaryotic initiation factor 2 (eIF2α) to decrease protein translation [40–42] and/or by inducing apoptosis of infected cells [43, 44].

Apart from its traditional antiviral roles, our group is more interested in the role of PKR in regulating inflammation. For instance, PKR can be activated during inflammation, as illustrated by an increase of PKR phosphorylation in macrophages following CD40 ligation [45] and when stimulated by toll-like receptor (TLR) ligands [46]. Furthermore, PKR shows crosstalk with inflammatory pathways such as c-Jun-N-terminal kinase (JNK) [46, 47], mitogen-activated protein kinase (MAPK) [48], nuclear factor-kappa B (NF-κB) [47, 49], signal transducers and activators of transcription 1 (STAT1) [50], interferon-regulatory factor-1 (IRF-1) [49], and inflammasome [51]. It can affect cytokine production and release from cultured fibroblasts [47, 48], macrophages [46, 51], and mixed glia/neuron co-cultures [52]. On the other hand, genetic deletion of PKR in mice attenuates plasma IL-6 and IL-12 increases triggered by LPS [48]. Until recently, PKR has also been shown to control neuroinflammatory changes in animal models of viral encephalitis [53] and excitotoxic injury [54].

Since systemic inflammation triggers neuroimmune activation and sickness, and that PKR modulates inflammation, the purpose of this study is to investigate whether PKR can affect neuroimmune responses and sickness induced by systemic inflammatory challenge. Wild-type (WT) and PKR−/− mice were subcutaneously injected with live *Escherichia coli* (*E. coli*) or vehicle. As predicted, deficiency of PKR diminished peripheral inflammatory responses to *E. coli*. However, to our surprise, the loss of PKR did not decrease sickness. Instead, PKR−/− mice displayed several behavioral components of sickness (reduced burrowing, exploratory deficits, and social withdrawal) that were not observed in WT mice. Moreover, these altered sickness behaviors were unlikely to be caused by exaggerated neuroimmune activation or increased bacterial load, because both strains of mice showed similar neuroimmune responses and bacterial titers throughout the course of infection. As systemic inflammation can activate the hypothalamic-pituitary-adrenal (HPA) axis which can potentially regulate sickness [55–60], we further asked whether PKR modulates HPA axis activation following *E. coli* challenge. Both strains of mice exhibited similar changes in plasma corticosterone levels post-infection. Nevertheless, PKR−/− mice displayed a delayed induction of corticotrophin-releasing hormone (CRH) in the hypothalamus as compared to WT mice, suggesting that the loss of PKR may postpone the CRH response to systemic inflammation. Taken together, our findings show that (1) deficiency of PKR could alter *E. coli*-induced sickness behaviors and (2) this was not because of exacerbated neuroimmune activation, (3) increased bacterial load, or (4) dysregulation of the corticosterone response. However, knockout of PKR could delay the CRH response to systemic inflammation, and this may possibly affect sickness.

## Methods

### Animal husbandry

All animal procedures were approved by the Committee on the Use of Live Animals in Teaching and Research (CULATR) of the University of Hong Kong (HKU). Wild-type C57BL/6 mice were purchased from the Laboratory Animal Unit (LAU) of the LKS Faculty of Medicine in HKU. Breeders of PKR−/− mice having targeted disruption in exons 2 and 3 of the PKR gene [61] were generously given by Prof. Bryan Williams. These mice were maintained on a C57BL/6 background prior to their arrival at our laboratory. Genome-wide single nucleotide polymorphism (SNP) scanning (The Jackson Laboratory, Bar Harbor, USA) also verified that 99 % of the genetic makeup of PKR−/− mice is of C57BL/6. All mice were bred under a specific pathogen-free (SPF) environment maintained at 24–26 °C and under a 12:12 h light:dark cycle (lights on 08:00–20:00) in LAU. Mice were used when they reached maturity at 3 months old, and only females were included to limit fighting. Individual variability in the estrous cycle was not controlled in animal husbandry. Mice were group caged (4–5 mice per cage) and provided with food and water ad libitum.

### Bacterial culture

*E. coli* (ATCC 15746, American Type Collection, Manassa, VA) was cultured as previously described [62–64]. Briefly, after reconstituting in 35-ml brain heart infusion (BHI) broth (BD Diagnostic Systems, NJ, USA), the culture was incubated overnight at 37 °C, 5 % CO_2_. On the next day, it was supplemented with glycerol (10 % *v*/*v*), aliquoted, frozen, and stored at −80 °C. These aliquots served as glycerol stocks. Typically, on the day before injection, 0.5 ml of glycerol stock was used to inoculate 35 ml BHI and grown overnight as described above. The bacterial concentration was quantified by measuring its absorbance at 595 nm and extrapolating from a previously determined standard curve. The bacteria were centrifuged at 4000×*g* for 30 min, the supernatant was removed, and the bacterial pellet was re-suspended in sterile phosphate buffered saline (PBS).

### Experimental paradigm

Four days before the injection, mice were transferred from LAU to an isolated behavioral testing room that was maintained at 24–26 °C and under a 10:14 h light:dark cycle (lights on 09:00–19:00). A small area of hair, i.e., 1 × 1 cm, was shaved from the back (i.e., just caudal to the ears) of each mouse to facilitate inspection of local inflammation. Mice were then singly caged, and allowed to habituate for 4 days in the behavioral room. On the day after the habituation period, mice were either injected with 0.5 ml sterile PBS (vehicle) or 8 × 10^8^ colony-forming unit (CFU) *E. coli*. This dose was chosen based on our preliminary observations that it can produce obvious signs of sickness (e.g., anorexia, decreased activity) in mice for several days without causing severe mortality (mortality rate was below 1 %) or extreme weight loss (weight loss was less than 10 % of the starting body weight). All injections took place at 11:00–12:00, and mice were monitored for the next 120 h.

### Food consumption

The amount of food given to each mouse (placed on the cage top) was pre-weighed, and the amount remaining 24 h later was weighed again. The amount of food consumed in a day was calculated by the difference between the two masses. Any food material that was not consumed in the previous day was discarded to minimize spill over to the next day.

### Rotarod test

The rotarod test was performed as previously described but with some minor modifications [65]. Briefly, the apparatus consisted of a horizontal rotatable rod (10 cm long, 6 cm in diameter) surrounded by vertical boards to prevent animal escape. A padding material (a stack of plastic bags) was placed at the bottom of the setup to minimize animal injury when the animal falls from the rod. A mouse was placed with its front paws just touching the rotating rod and immediately released. The speed of rotation was gradually accelerated, and the time of which the mouse stayed on the rod before falling was recorded. Mice staying for more than 3 min were counted as staying for 3 min. Each mouse was given three trials each time, and the average of the three readings was used for data analysis. Mice were trained for the rotarod test on two different days of the habituation period to acclimatize them to the procedure, and tested at 24 and 96 h after the injection.

### Burrowing

Burrowing is a rodent-specific behavior, and this behavior has been used to study sickness [11, 27, 28]. This setup consisted of an opaque and hollow plastic tube (6 × 6 × 15 cm), sealed at one end and with the other end being tilted at an angle such that it could stand 1 cm above the cage floor. It was filled with a bedding material (~60 g), and together they were pre-weighed. The tube was then introduced into the cage, and the mouse was allowed to burrow while the experimenter remained outside the room. One hour after testing, the tube and its contents were weighed again, and the amount of bedding material burrowed was calculated by the difference between the two readings. Mice were trained to burrow during the habituation period to limit inter-individual variation in baseline burrowing activity and were tested every 24 h after the injection.

### Open field test

The open field test [66] was conducted in a 40 cm (L) × 30 cm (W) × 40 cm (H) white plastic arena, with a floor that was gridded into 12 equal 10 × 10 cm squares. A mouse facing at a corner of the arena, and having its front paws just touching the arena floor, was quickly released. It was allowed to explore the open field for 3 min, during which its behavior was videotaped. Testing was performed under quiet and dim lighting, and the experimenter remained outside the behavioral room throughout the test. The number of lines crossed by the mouse in this novel environment was determined from the video, and validated by an independent experimenter. Testing was performed at 120 h post-injection.

### Object investigation test

Immediately after the open field test, the mouse was temporarily returned to its home cage, and two identical transparent plastic cages, i.e., 8 cm (L) × 6 cm (W) × 12 cm (H) and having holes on its sides, were placed one on each side (6 cm side) of the arena. The mouse was introduced back into the arena, with its head facing the wall of one of the 8-cm sides and its front paws just touching the floor, and immediately released. It was allowed to explore the two transparent cages for 3 min, during which it was videotaped. The experimenter remained outside the room. The amount of time the mouse spent investigating both novel cages was determined from the videotape and validated by another independent experimenter. A mouse was regarded as interacting with the cage if it (1) physically contacted with the cage or if (2) its nose was 2 cm near the cage. Any accidental bumping of the mouse to the cage by its limbs or its body was not regarded as interaction. One *E. coli*-injected PKR−/− mice jumped onto a cage and remained there for the whole period, and hence it was not included in the data analysis.

### Social interaction test

The social interaction test was conducted similarly as reported but with some modifications [67]. Immediately following the object investigation test, the mouse was returned back to its home cage, and a novel female C57BL/6 mouse (~3 weeks old) was put into one of the two empty cages. The subject mouse was introduced back into the arena in the same way as above. The behavior of the mouse was recorded for the next 3 min. The time that the subject mouse spent interacting with the empty cage and the time that it spent interacting with the social cage were both deduced from the video and validated by an independent experimenter. Five mice jumped onto a cage and were rejected for data analysis. After the test, the entire apparatus was washed with running tap water, wiped with towel, and allowed to dry before testing the next mouse. The same novel mouse was used across all subject mice.

### Total RNA extraction, genomic DNA digestion, and reverse transcription

Mice were sacrificed with an overdose of sodium pentobarbital (100 mg/kg). The liver and brain were quickly collected, and the hypothalamus (within Bregma 0.86 to −2.7 mm) and hippocampus (within Bregma −0.82 to −3.8 mm) were isolated on ice. Total RNA was extracted using TRI Reagent® RT (Molecular Research Centre Inc, Cincinnati, USA) according to the manufacturer’s protocol. The RNA concentrations in the samples were quantified by NanoDrop 2000c (Thermo Scientific, Wilmington, USA). All RNA samples had A260/A280 ratios of 1.8–2.0 and showed distinct bands for the 28S and 18S rRNA subunits on a gel. To remove any contaminating genomic DNA, 4 μg total RNA from each sample was treated with DNA-*free*™ DNA Removal Kit (Life Technologies, Carlsbad, USA) in a 22-μl reaction volume, and 8 μl of the reaction (~1.45 μg total RNA) was taken for reverse transcription by SuperScript™ III First-Strand Synthesis system (Life Technologies, Carlsbad, USA). cDNA samples were 10× diluted in DEPC-treated water.

### Real-time PCR

Two microliters of diluted cDNA was amplified in triplicate with SYBR Premix Ex Taq™ II kit, Perfect Real Time (Takara Bio Inc, Shiga, Japan) and employing the MyiQ™2 two-color Real-Time PCR detection System (Bio-Rad, Hercules, USA). The general PCR protocol was as follows: 95 °C for 120 s, 95 °C for 15 s, annealing temperature (refer to Table 1) for 30 s, and 72 °C for 10–30 s (see Table 1) for 45 cycles. The specificity of PCR was confirmed by ensuring that there was only one peak in the melting curve analysis and only one band matching to the size the PCR product on a gel. All PCR amplification efficiencies were 90–110 %. The expression level of each mouse gene was extrapolated from the standard curve and normalized to the expression level of a housekeeping gene glyceraldehyde 3-phosphate dehydrogenase (GAPDH). Our preliminary results indicated that GAPDH expression was unaffected by genotype or by *E. coli* in the brain and liver. Results for the mouse genes were reported as relative mRNA expression. Results of 16S ribosomal DNA (rDNA) do not require the normalization step and were reported as fold of the WT PBS group.

**Table 1:** The PCR conditions for IL-1β, TNF-α, IL-6, COX-2, iNOS, CRH, GAPDH, and 16S rDNA

Gene	Primer sequences	Working primer concentration (μM)	Annealing temperature (°C)	Extension time (s)	Amplicon size (base pair)
IL-1β (118130747)	F: 5′-CCTCCTTGCCTCTGATGG-3′	0.4	60	10	99
	R: 5′-AGTGCTGCCTAATGTCCC-3′				
TNF-α (518831586)	F: 5′-CCCCAGTCTGTATCCTTCT-3′	0.4	59	30	106
	R: 5′-ACTGTCCCAGCATCTTGT-3′				
IL-6 (13624310)	F: 5′-GGCAATTCTGATTGTATG-3′	0.4	56	30	208
	R: 5′-CTCTGGCTTTGTCTTTCT-3′				
COX-2 (118130137)	F: 5′-GATGACTGCCCAACTCCC-3′	0.25	60	10	191
	R: 5′-AACCCAGGTCCTCGCTTA-3′				
iNOS (146134510)	F: 5′-AAACGCTTCACTTCCAATG-3′	0.4	61	30	296
	R: 5′-CAATCCACAACTCGCTCC-3′				
CRH (292781723)	F: 5′-GTGCGGGCTCACCTACCAA-3′	0.4	57	10	107
	R: 5′-AGGCAGGCAGGACGACAGA-3′				
GAPDH (576080554)	F: 5′-ATTCAACGGCACAGTCAA-3′	0.4	56	10	77
	R: 5′-CTCGCTCCTGGAAGATGG-3′				
16S rDNA (J01859.1)	F: 5′-GCAAGCGGACCTCATAAA-3′	0.25	54	20	100
	R: 5′-ATTCACCGTGGCATTCTG-3′				

The real-time PCR conditions for IL-1β, TNF-α, IL-6, COX-2, iNOS, CRH, GAPDH, and 16S rDNA. Accession numbers from the Genebank are shown in brackets.

*COX-2* cyclooxygenase-2, *CRH* corticotrophin-releasing hormone, *iNOS* inducible nitric oxide synthase, *TNF-α* tumor necrosis factor-α, *IL* interleukin

### Assessment of blood neutrophil percentage

An increase of blood neutrophil granulocytes can serve as an indicator of systemic inflammation [68]. To assess this, whole blood was collected from mice by making a tail nick. Twenty microliters of blood was spotted onto a glass slide to produce a blood smear, and three blood smears were prepared per mouse. Slides were fixed in methanol for 30 s and allowed to dry under room temperature. They were stained with 5 % Giemsa stain, pH 6.8 (Medical Chemical Corporation, Torrance, USA) for 20 min and briefly washed in deionized water. Next, they were dehydrated in a graded series of ethanol, cleared in toluene, mounted with Permount (Sigma Aldrich, St. Louis, USA), and coverslipped. Slides were viewed under a 40× objective of a Zeiss Axiophot microscope (Carl Zeiss, Thornwood, USA) connected to a SPOT RT3™ camera (SPOT Imaging Solutions, Sterling Heights, USA). Ten bright view images were captured at the “zone of morphology” in each slide, and three slides per mouse. The numbers of neutrophils and leukocytes were manually counted. Neutrophil % was calculated by Neutrophil % = (Average no. of neutrophils/Average no. of leukocytes) × 100 %.

### Determination of bacterial titer

Bacterial titers at the injected site and in blood were quantified every 24 h by microbiological plate count and by real-time PCR for bacterial 16S rDNA. Briefly, lavage fluid was collected by subcutaneously injecting 1 ml of sterile phosphate buffered saline (PBS) at the injection site and immediately withdrawing ~200 μl using another pair of sterile needle and syringe. Blood was obtained by making a tail nick as described above and supplemented with sterile disodium EDTA (3.6 mM) for anti-coagulation. For the plate count method, blood and lavage samples were serially diluted with sterile PBS. The diluted samples were spread onto pre-warmed lysogeny broth (LB) agar plates (BD Diagnostic Systems, NJ, USA) and incubated at 37 °C. After 24 h, the number of colonies was counted by two independent observers, and the corresponding bacterial titer in the undiluted sample was calculated. The 16S rDNA method was performed as previously described but with a few minor modifications [69]. In brief, 20 μl of lavage fluid and 50 μl of anti-coagulated blood were respectively treated with 25 and 65 U of mutanolysin (Sigma Aldrich, St. Louis, USA) at 37 °C for 30 min. Genomic DNA was purified from the mutanolysin-treated samples using the QIAamp DNA Mini kit (Qiagen Inc., Valencia, CA) based on the manufacturer’s protocol and eluted in 100 μl of double distilled water. Two microliters of the elution was used for real-time PCR for 16S rDNA as described before.

### TNF-α and corticosterone ELISA

Mice were sacrificed with an overdose of sodium pentobarbital (100 mg/kg) at 4 h (16:00) and 48 h (12:00). Heparinized blood was collected by cardiac puncture, centrifuged, and the plasma was removed and stored at −80 °C until analysis. TNF-α and corticosterone levels were determined using Mouse TNF-alpha DuoSet ELISA Kit (R&D systems Inc, Minneapolis, Canada) and Corticosterone EIA Kit (Cayman Chemical Company, Ann Arbor, USA), respectively, according to the manufacturers’ instructions. For the ELISA of corticosterone, plasma samples were first 200× diluted in assay buffer. The detection limit of the TNF-α ELISA assay was 31 pg/ml, and that of the corticosterone ELISA assay was 30 pg/ml (i.e., 6 ng/ml in the undiluted sample).

### Data and statistical analysis

Statistical analysis was conducted by SigmaStat® 3.0 (Jandel Scientific, San Rafael, USA). Data for food consumption, burrowing, and the rotarod test were analyzed by two-way repeated measures of analysis of variance (ANOVA) with genotype (WT *E. coli* v.s. PKR−/− *E. coli*) and time as the main factors. If a significant interaction effect could be detected, Bonferroni post hoc comparison was performed. Additionally, selected time points for food consumption and the rotarod test were analyzed by one-way ANOVA followed by Student-Newman-Keuls post hoc comparison. The open field test, object investigation test, and social interaction test were replicated in two independent batches of experiments (including all four treatment groups in each batch). For the open field and object investigation tests, data were first expressed as fold of the WT PBS group of the same batch of experiment before both batches were combined for data analysis. They were then analyzed by one-way ANOVA followed by Student-Newman-Keuls post hoc comparison. For the social interaction test, data from the same experiment were expressed as fold of interaction with the social cage of the WT PBS group, and data from both batches were pooled. Interaction effect between experimental group (WT or PKR−/− mice injected with PBS or *E. coli*) and cage (social v.s. empty) was analyzed by two-way ANOVA, followed by, if significant, Bonferroni post hoc comparison. Data including all four experimental groups (WT PBS, WT *E. coli*, PKR−/− PBS, PKR−/− *E. coli*) for real-time PCR, neutrophil %, and ELISA were analyzed by one-way ANOVA followed by Student-Newman-Keuls post hoc comparison. Additionally, two-tailed *t* tests were performed to analyze for potential differences in baseline corticosteroid levels across time (4 h WT PBS v.s. 48 h WT PBS; 4 h PKR−/− PBS v.s. 48 h PKR−/− PBS). Data of bacterial plate count and 16S rDNA were positively skewed and were thus log transformed [70]. Transformed data were analyzed by two-way ANOVA with genotype (WT *E. coli* v.s. PKR−/− *E. coli*) and time as the main factors. All values were represented as mean ± standard error from the mean (SEM), and results were considered significantly different if *p* < 0.05.

## Results

### PKR−/− mice displayed altered *E. coli*-induced sickness behaviors

Systemic inflammation induces both core (e.g., fever, anorexia, motor impairments) and behavioral (e.g., social withdrawal, reduced exploration, anhedonia, cognitive deficits) components of sickness [5, 19, 71]. As PKR has been known to regulate inflammation [36, 72], we evaluated whether loss of PKR would also affect these two components of sickness.

**Fig. 1:**
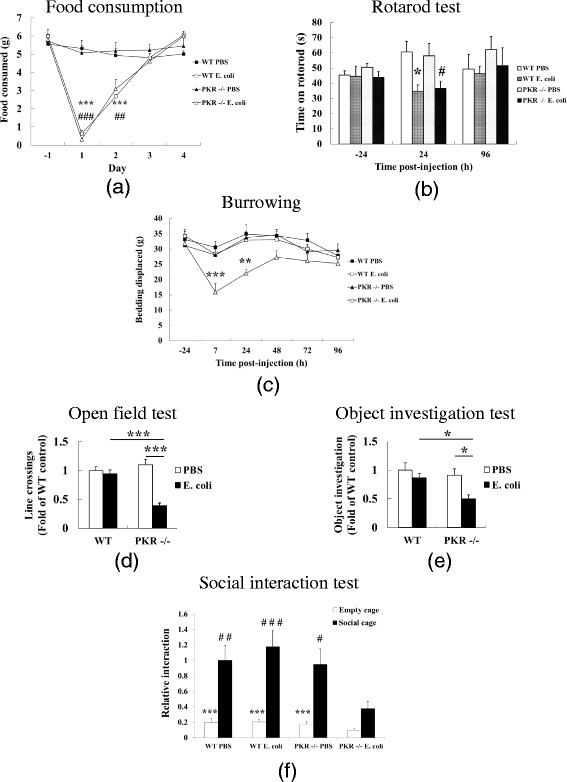
Effects of PKR deficiency on *E. coli*-induced sickness. WT and PKR−/− mice were subcutaneously challenged by *E. coli* or PBS, and sickness responses were monitored for the next 120 h. Infection of *E. coli* led to similar decreases in food consumption (**a**) and rotarod test performance (**b**) in WT and PKR−/− mice. *p < 0.05, ***p < 0.001, WT *E. coli* versus WT PBS; +*p* < 0.05, +++*p* < 0.001, PKR *E. coli* versus PKR PBS; *n* = 8 per group. However, *E. coli* suppressed burrowing in PKR−/− mice but not in WT mice (**c**). **p < 0.01, ***p < 0.001, PKR−/− *E. coli* versus PKR−/− PBS, *n* = 8 per group. Furthermore, at 120 h post-injection, *E. coli* downregulated open field (**d**) and object investigation (**e**) activities in PKR−/− mice but not in WT mice. *p < 0.05, ***p < 0.001; *n* = 13–16 per group. Likewise, *E. coli* decreased social interaction in PKR−/− mice but not in WT mice at 120 h (**f**). ***p < 0.001, empty cage versus social cage of the same experimental group; #*p* < 0.05, ##*p* < 0.01, ###*p* < 0.001 versus interaction with the social cage of the PKR−/− *E. coli* group; *n* = 12–15 per group

Deficiency of PKR did not affect the core components of sickness. For example, WT and PKR−/− mice displayed similar reductions in food consumption after *E. coli*, and these changes were gradually restored by day 3 (Fig. 1a). Two-way repeated measures ANOVA of the *E. coli*-treated groups did not indicate any interaction effect (*F* = 0.749, df 4, 60, *p* = 0.563) between genotype (WT *E. coli* or PKR−/− *E. coli*) and the time post-injection, implying that the genotype had no effect on the anorexic responses across time. As further supportive evidence, one-way ANOVA followed by Student-Newman-Keuls post hoc comparison showed that *E. coli* was able to decrease (*p* < 0.001) food consumption in WT mice (WT PBS v.s. WT *E. coli*) and PKR−/− mice (PKR−/− PBS v.s. PKR−/− *E. coli*) on day 1 and day 2, but no difference (*p* > 0.05) could be found between the *E. coli*-challenged groups on both days. Likewise, both strains of mice showed similar motor impairments in the rotarod test following *E. coli* (Fig. 1b). Two-way repeated measures ANOVA of the *E. coli*-challenged groups did not show any interaction between genotype and time (*F* = 0.0753, df 2, 27, *p* = 0.0928). Moreover, one-way ANOVA with Student-Newman-Keuls post hoc test indicated that *E. coli* decreased motor performance (*p* < 0.05) in WT and PKR−/− mice at 24 h, but again there was no difference (*p* = 0.822) between the *E. coli*-treated groups.

Although the core components of sickness were similar between WT and PKR−/− mice, the behavioral components of sickness were altered in PKR−/− relative to WT mice. As shown in Fig. 1c, *E. coli* reduced burrowing in PKR−/− mice but not in WT mice. Two-way repeated measures ANOVA of the *E. coli*-challenged groups indicated a main effect of genotype (*F* = 9.002, df 1, 75, *p* = 0.009) and an interaction between genotype and time (*F* = 2.796, df 5, 75, *p* = 0.023), suggesting that the effect of genotype on the burrowing deficits depended on the time post-injection. Bonferroni post hoc comparison showed that the PKR−/− *E. coli* group had significantly lesser burrowing activity than the WT *E. coli* group at 7 h (*p* < 0.001) and 24 h (*p* = 0.001). In addition, at 120 h post-injection, a time when the core components of sickness had disappeared in both strains of mice, PKR−/− mice still displayed reduced exploratory activities in the open field test (Fig. 1d) and object investigation test (Fig. 1e). One-way ANOVA followed by Student-Newman-Keuls post hoc test showed that *E coli* significantly decreased (*p* < 0.05) the exploratory activities of PKR−/− mice (PKR−/− PBS v.s. PKR−/− *E. coli*) in both tasks and that the PKR−/− *E. coli* group also explored significantly lesser as compared to the WT *E. coli* group. Finally, we found that *E. coli* triggered social withdrawal in PKR−/− mice but not in WT mice at 120 h (Fig. 1f). Two-way ANOVA with cage (social v.s. empty) and experimental grouping (WT PBS/WT *E. coli*/PKR−/− PBS/PKR−/− *E. coli*) as the main factors indicated a main effect of cage (*F* = 60.489, df 1, 96, *p* < 0.001) and an interaction between cage and experimental group (*F* = 3.044, df 3, 96, *p* < 0.033). Hence, the effect of the social cage depended on the experimental group. Bonferroni post hoc comparison found significant differences (*p* < 0.001) between social v.s. empty cages in the WT PBS, WT *E. coli*, and PKR−/− PBS groups, but not in the PKR−/− *E. coli* group (*p* = 0.099). The investigation of the social cage in the PKR−/− *E. coli* group was also significantly lesser than that of the other three experimental groups (*p* < 0.05).

### PKR−/− mice displayed reduced peripheral inflammatory responses to *E. coli*

A previous study has demonstrated that genetic deficiency of PKR can attenuate plasma cytokine increases following systemic LPS challenge [48]. Hence, it would be reasonable to postulate that loss of PKR should similarly reduce peripheral inflammatory changes after a subcutaneous injection of *E. coli*. However, since only PKR−/− mice developed observable behavioral components of sickness, we first questioned whether genetic deletion of PKR would unexpectedly promote peripheral inflammation in response to an *E. coli* infection.

**Fig. 2:**
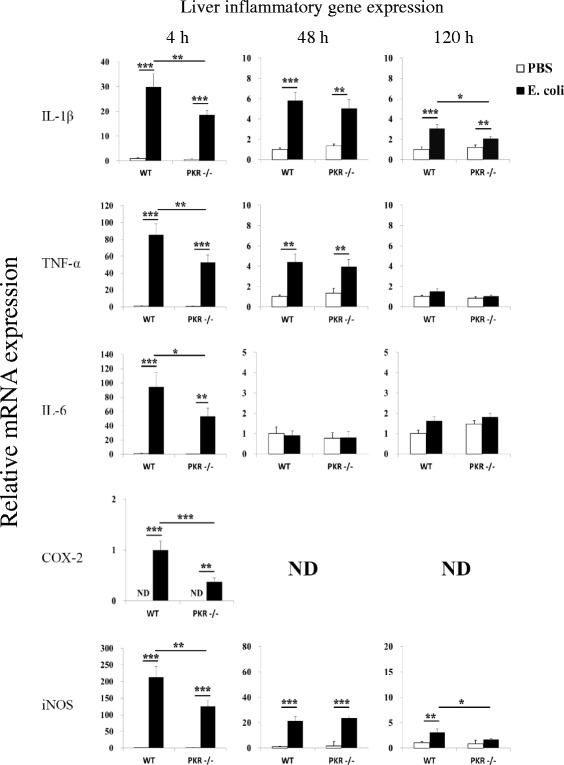
Relative mRNA expression profiles of IL-1β, TNF-α, IL-6, COX-2, and iNOS in the liver. Loss of PKR inhibited inflammatory gene expression in the liver triggered by *E. coli*, particularly at 4 h. *n* = 7–8 per group at 4 h, *n* = 6–8 per group at 48 h, and *n* = 9–11 per group at 120 h. *ND* not detectable; *p < 0.05, **p < 0.01, ***p < 0.001

**Fig. 3:**
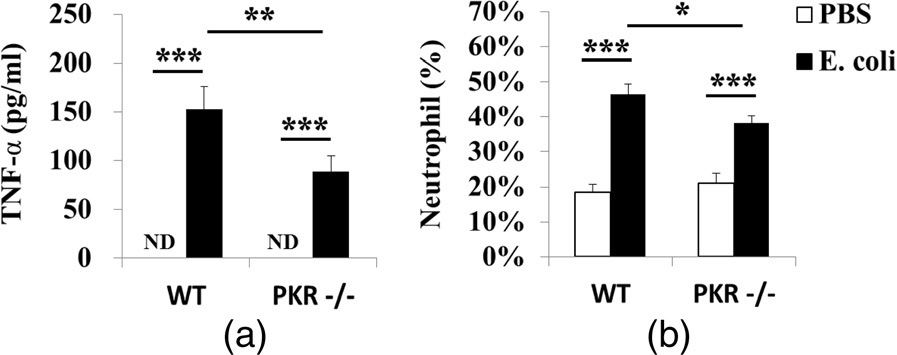
Deficiency of PKR led to smaller increases of plasma TNF-α and neutrophil %. Peripheral blood was collected at 4 h for TNF-α ELISA (**a**) and at 120 h (**b**) for peripheral leukocyte count after the injection of PBS or *E. coli. n* = 8–10 per group in **a**, and *n* = 13–16 per group in **b**. *ND* not detectable; *p < 0.05, **p < 0.01, ***p < 0.001

Consistent with the earlier study [48], PKR−/− mice showed diminished peripheral inflammatory responses to *E. coli*. Genetic deletion of PKR suppressed inflammatory gene induction (IL-1β, TNF-α, IL-6, cyclooxygenase (COX)-2, iNOS) in the liver (Fig. 2), where the highest density of tissue macrophages, i.e., Kupffer cells, reside in the body [73]. Particularly at 4 h, post hoc Student-Newman-Keuls test after one-way ANOVA showed that *E. coli* upregulated (*p* < 0.01) all five inflammatory genes in both strains of mice, but the induction in PKR−/− mice was significantly lower (*p* < 0.05) than that of WT mice. At 48 h, the levels of IL-1β, TNF-α, and iNOS still remained elevated (*p* < 0.01) by *E. coli*, but there was no significant difference (*p* > 0.05) between the *E. coli*-treated groups. At 120 h, even smaller increases (*p* < 0.05) of IL-1β (WT and PKR−/− mice) and iNOS (WT mice only) were detected, and the expression of these genes were significantly lower (*p* < 0.05) in the PKR−/− *E. coli* group than in the WT *E. coli* group. In line with these findings, deficiency of PKR led to a lesser increase of plasma TNF-α at 4 h after *E. coli* challenge (Fig. 3a). In the PBS-injected groups, plasma TNF-α levels were below the detection limit (<31 pg/ml) of the ELISA assay. One-way ANOVA followed by Student-Newman-Keuls post hoc test indicated that *E. coli* elevated plasma TNF-α in WT mice (WT PBS v.s. WT *E. coli*; *p* < 0.001) and PKR−/− mice (PKR−/− PBS v.s. PKR−/− *E. coli*; *p* < 0.001) and that the PKR−/− *E. coli* group had a lower level of TNF-α than the WT *E. coli* group (WT *E. coli* v.s. PKR−/− *E. coli*; 152 ± 24 v.s. 88 ± 17 pg/ml; *p* = 0.003). Finally, an elevation of neutrophil granulocytes in blood can serve as an index of systemic inflammation [68]. We found that deficiency of PKR in mice triggered a smaller increment of neutrophil % at 120 h post-injection (Fig. 3b). One-way ANOVA followed by Student-Newman-Keuls post hoc comparison showed that *E. coli* upregulated neutrophil % in both WT mice (WT PBS v.s. WT *E. coli*; 18.5 ± 2.1 v.s. 46.7 ± 3.1 %; *p* < 0.001) and PKR−/− mice (PKR−/− PBS v.s. PKR−/− *E. coli*; 21.2 ± 2.7 v.s. 38.1 ± 2.2 %; *p* < 0.001), and also a lower neutrophil % was seen in the PKR−/− *E. coli* group than in the WT *E. coli* group (*p* = 0.028).

### Neuroimmune responses in WT and PKR−/− mice were similar

Since peripheral inflammatory changes were suppressed in PKR−/− mice, we asked whether neuroimmune responses would also be dampened in PKR−/− mice. Therefore, we assessed inflammatory gene expression in the hypothalamus and hippocampus after *E. coli* challenge in WT and PKR−/− mice. These two brain regions were selected for analysis because of their high expression for cytokine receptors in the brain [74–76] and that they are important in the control of the sickness [2]. To our surprise, we found that both strains of mice displayed mostly similar increases of inflammatory genes at both brain regions.

**Fig. 4:**
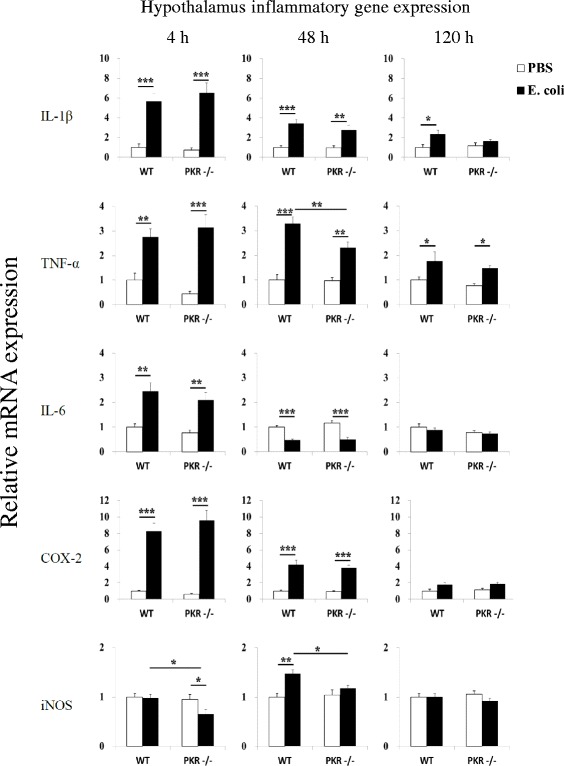
Relative mRNA expression profiles of IL-1β, TNF-α, IL-6, COX-2, and iNOS in the hypothalamus. The loss of PKR had little effect on inflammatory gene expression in the hypothalamus after *E. coli* challenge. *n* = 7–8 per group at 4 h, *n* = 6–8 per group at 48 h, and *n* = 9–11 per group at the 120 h time point. *p < 0.05, **p < 0.01, ***p < 0.001

At 4 h in the hypothalamus (Fig. 4), *E. coli* significantly upregulated mRNA expression for IL-1β, TNF-α, IL-6, and COX-2 in WT and PKR−/− mice (*p* < 0.05, one-way ANOVA followed by Student-Newman-Keuls post hoc test), but no significant difference could be detected (*p* > 0.05) between the *E. coli*-challenged groups for all four genes. On the other hand, *E. coli* led to a small drop (~30 %, *p* < 0.05) in iNOS mRNA expression in PKR−/− mice (PKR−/− PBS v.s. PKR−/− *E. coli*), and the PKR−/− *E. coli* group also showed significantly lesser expression of iNOS (*p* < 0.05) than the WT *E. coli* group. At 48 h, *E. coli* was still able to elevate IL-1β, TNF-α, and COX-2 expression (*p* < 0.01) in both genotypes of mice, and it also increased iNOS expression (*p* < 0.01) in WT mice. Among these genes, the PKR−/− *E. coli* group displayed significantly lower expression for TNF-α (*p* < 0.01) and iNOS (*p* < 0.05) than the WT *E. coli* group. IL-6 mRNA was similarly reduced by *E. coli* in both strains of mice (*p* < 0.001), and no significant difference was found between the *E. coli*-treated groups. At 120 h, small increases of TNF-α (both genotypes, *p* < 0.05) and IL-1β (WT mice only, *p* < 0.05) could still be observed, but no significant difference (*p* > 0.05) could be found among the *E. coli*-treated groups for both genes.

**Fig. 5:**
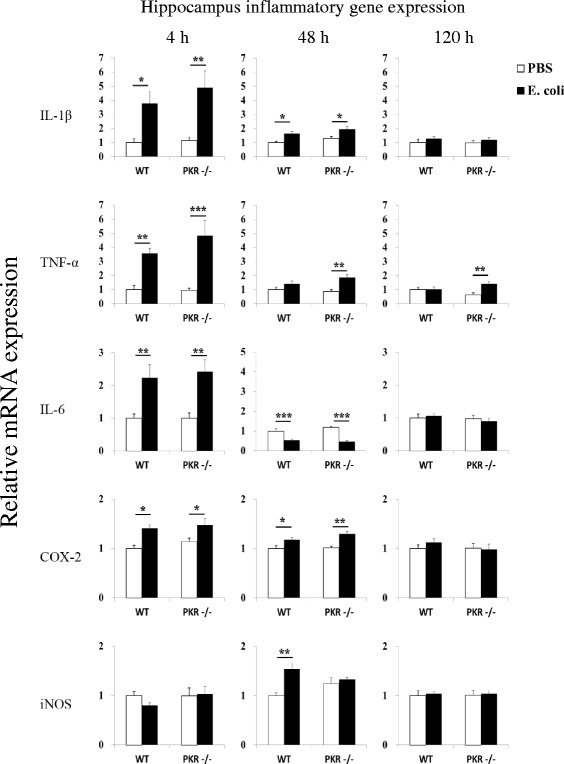
Relative mRNA expression profiles of IL-1β, TNF-α, IL-6, COX-2, and iNOS in the hippocampus. The deficiency of PKR had minimal effect on inflammatory gene expression in the hippocampus after *E. coli* challenge. *n* = 7–8 per group at 4 h, *n* = 6–8 per group at 48 h, and *n* = 9–11 per group at 120 h. *p < 0.05, **p < 0.01, ***p < 0.001

Similarly, at 4 h in the hippocampus (Fig. 5), *E. coli* upregulated the mRNA expression of IL-1β, TNF-α, IL-6, and COX-2 (*p* < 0.05) in WT and PKR−/− mice, but no significant difference (*p* > 0.05) was found between the *E. coli*-challenged groups of the two strains of mice. Likewise, at 48 h, there were still small increases (i.e., less than onefold, *p* < 0.05) in the mRNA expression of IL-1β and COX-2 (WT and PKR−/− mice), TNF-α (PKR−/− mice only), and iNOS (WT mice only), but none of these inflammatory genes displayed any significant difference (*p* > 0.05) between the *E. coli*-treated groups. At the same time point, IL-6 expression was decreased in both genotypes by *E. coli* (*p* < 0.001), but the *E. coli*-treated groups did not differ in IL-6 expression. Finally, at 120 h, only a small increase of TNF-α (~40 %, *p* < 0.01) was observed in PKR−/− mice after *E. coli*, but this also did not reach statistical difference (*p* = 0.11) when compared to the WT *E. coli* group.

### Bacterial titers were similar between WT and PKR−/− mice

Given that loss of PKR in mice reduced peripheral inflammatory responses but at the same time led to more observable behavioral components of sickness, we questioned whether PKR deficiency had exaggerated bacterial load. By performing standard microbiological plate counts and real-time PCR for bacterial 16S rDNA, we found that the changes of bacterial titers were similar between the two strains of mice at the injection site (i.e., lavage fluid) and in blood throughout the course of infection.

**Fig. 6:**
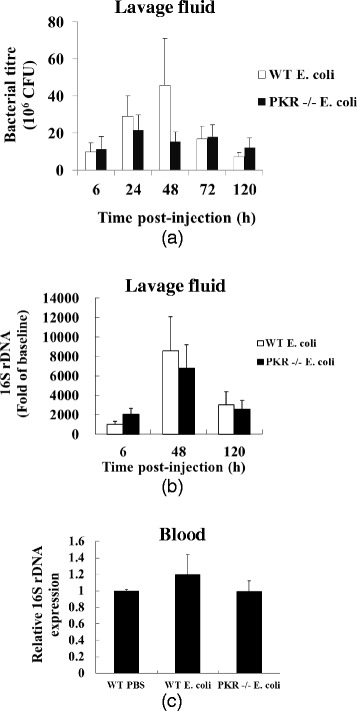
Loss of PKR did not enhance bacterial load. Lavage fluid (injection site) and blood were sampled at the indicated time points. In (**a**), serially diluted lavage samples from *E. coli*-challenged mice were plated onto bacterial agar plates and incubated at 37 °C for 24 h. The numbers of colony-forming units (CFU) were similar between WT and PKR−/− mice throughout the time points; n = 17–19 per group. There was no CFU detected in the lavage fluid from the PBS-injected mice. In (**b**), purified lavage fluid from *E. coli*-infected animals were tested for their bacterial 16S rDNA levels, and expressed as fold of baseline (i.e., PBS-injected animals). Similar levels of 16S rDNA were observed between WT and PKR−/− animals; n = 13–18 per group. In (**c**), anti-coagulated blood was collected from mice at 48 h, purified, and assessed for 16S rDNA. No significant increase of 16S rDNA could be found in the purified blood samples of the two infected groups as compared to the WT PBS group; n = 3 per group. We also could not culture any bacterial colonies from blood sampled at this time point

For the plate count method, we observed that lavage fluid from the PBS-injected mice of both strains did not produce any colonies, thus ruling out the possibility of sample contamination during the procedure. However, lavage fluid from the *E. coli*-injected mice gave rise to numerous bacterial colonies (Fig. 6a). Two-way ANOVA of the log transformed data indicated a main effect of time (*F* = 4.206, df 4149, *p* = 0.003) on the level of colony-forming units (CFU), but no interaction could be detected between genotype (WT *E. coli* v.s. PKR−/− *E. coli*) and time (*F* = 0.838, df 4, 149, *p* = 0.503). Hence, although bacterial titers varied with time, genotype had no effect on the change of bacterial titers across time. Moreover, we did not recover any bacterial colonies from the blood samples of the bacterial challenged mice at any of the time points included, suggesting that the *E. coli* infection did not spread into the blood circulation in either genotype.

Consistent to the plate count results, the levels of bacterial 16S rDNA in lavage fluid were also similar between WT and PKR−/− mice (Fig. 6b). Two-way ANOVA of the log transformed data indicated a main effect of time (*F* = 4.854, df 2, 75, *p* = 0.01), but there was no interaction between time and genotype (*F* = 0.671, df 2, 75, *p* = 0.542). Therefore, the change of 16S rDNA in lavage fluid across time points did not depend on the genotype. Since the level of 16S rDNA was the highest at 48 h, we also assessed the level of 16S rDNA in blood at this time point (Fig. 6c). One-way ANOVA did not indicate any difference between the WT PBS, the WT *E. coli*, and the PKR *E. coli* experimental groups (*F* = 0.547, df 2, 6, *p* = 0.605). Hence, such data also implied that the bacterial infection did not spread into the blood in both genotypes.

### PKR−/− mice exhibited a delayed induction of CRH in the hypothalamus

It is known that systemic inflammation can trigger hypothalamic CRH production and release [77] and that endogenous CRH production mediates inflammation-induced sickness [58, 78]. As PKR−/− mice demonstrated several behavioral components of sickness that were not observed in WT mice, we asked whether the CRH response in PKR−/− mice would be different from that of WT mice.

**Fig. 7:**
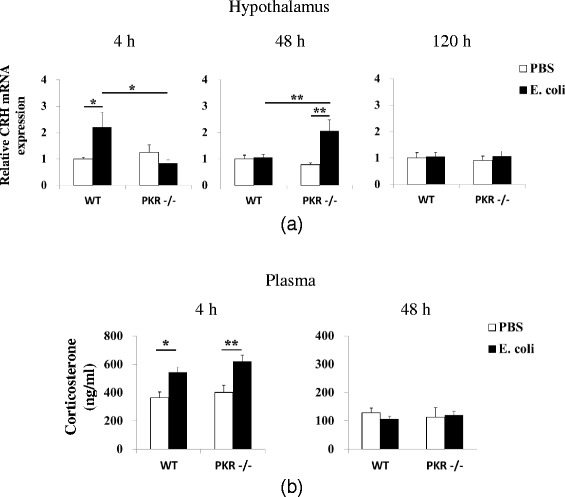
PKR deficiency delayed hypothalamic CRH induction but did not affect the plasma corticosterone response. Hypothalamic CRH mRNA expression (**a**) and plasma corticosterone levels (**b**) were assessed at indicated times after *E. coli* challenge. As shown in **a**, genetic deletion of PKR abolished the elevation of CRH by *E. coli* at 4 h but triggered a delayed increase in the PKR−/− *E. coli* group at 48 h. *n* = 7–8 per group at 4 h, *n* = 6–8 per group at 48 h, and *n* = 9–11 per group at 120 h. *p < 0.05, **p < 0.01. In **b**, *E. coli* caused similar increases of plasma corticosterone at 4 h (16:00) in WT and PKR−/− mice. By 48 h (12:00), corticosterone levels in the *E. coli*-treated groups had returned to control levels. *n* = 7–10 per group at 4 h, *n* = 5–7 per group at 48 h; *p < 0.05, **p < 0.01

We found that PKR−/− mice developed a delayed induction of CRH in the hypothalamus in response to *E. coli* challenge (Fig. 7a). At 4 h post-injection, *E. coli* significantly elevated CRH mRNA expression in WT mice (*p* = 0.021, one-way ANOVA followed by Student-Newman-Keuls post hoc comparison) but not in PKR−/− mice (*p* = 0.519), and a significant difference (*p* = 0.01) was also present between the *E. coli*-treated groups. In contrast, at 48 h post-injection, *E. coli* increased CRH expression only in PKR−/− mice (*p* = 0.003) but not in WT mice (*p* = 0.851). Furthermore, the PKR−/− *E. coli* group showed a significantly greater expression of CRH (*p* = 0.003) than the WT *E. coli* group. Finally, no detectable differences (*p* > 0.05) in CRH expression could be observed at 120 h.

### WT and PKR−/− mice showed similar changes in plasma corticosterone levels

We then asked whether PKR−/− mice would display a different profile for circulating corticosterone than WT mice in response to *E. coli*. In rodents, basal plasma corticosterone levels follow a circadian pattern, with low levels occurring during the morning and high levels in the evening [79]. This circadian pattern can be observed in WT and PKR−/− mice, with corticosterone levels being higher (two-tailed tests, *p* < 0.01) in the late afternoon (4 h/16:00) than in the morning (i.e., 48 h/12:00), i.e., 4 h WT PBS (363 ± 42 ng/ml) v.s. 48 h WT PBS (128 ± 17 ng/ml); 4 h PKR−/− PBS (402 ± 50 ng/ml) v.s. 48 h PKR−/− PBS (113 ± 33 ng/ml). Baseline corticosteroid levels did not differ between WT and PKR−/− mice (Fig. 7b). One-way ANOVA followed by Student-Newman-Keuls post hoc comparison showed no difference (*p* > 0.05) between the WT PBS and PKR−/− PBS groups at either time point. Immune challenge by *E. coli* led to similar changes in plasma corticosterone levels in both genotypes. At 4 h, *E. coli* increased corticosterone in both WT (WT PBS v.s. WT *E. coli*; 363 ± 42 v.s. 545 ± 35 ng/ml; *p* = 0.023) and PKR−/− mice (PKR−/− PBS v.s. PKR−/− *E. coli*; 402 ± 50 v.s. 622 ± 45 ng/ml; *p* = 0.004), but no difference was found between the *E. coli*-challenged groups (WT *E. coli* v.s. PKR−/− *E. coli*; *p* = 0.238). By 48 h, these increases had dissipated (WT PBS v.s. WT *E. coli*: 128 ± 17 v.s. 107 ± 10 pg/ml; PKR−/− PBS v.s. PKR−/− *E. coli*: 113 ± 33 v.s. 121 ± 13 ng/ml), and no significant difference (*p* > 0.05) could be detected between the experimental groups.

## Discussion

Systemic inflammation triggers neuroimmune activation, leading to sickness. In this study, we investigated the role of PKR, a serine-threonine kinase that is activated by immune challenge [45, 46] and regulates LPS-induced peripheral inflammatory responses [48], on neuroimmune responses and sickness following subcutaneous *E. coli* infection. While genetic deficiency of PKR in mice did not affect the core components of sickness (anorexia and motor impairments), it led to several behavioral components of sickness (decreased burrowing, exploratory deficits, and social withdrawal) that were not observed in WT mice (Fig. 1). Interestingly, such alteration in the behavioral components was not due to exacerbated inflammation, since the loss of PKR diminished peripheral inflammatory changes (Figs. 2 and 3), and it had minimal effect on the associated neuroimmune responses (Figs. 4 and 5). Likewise, bacterial titers (Fig. 6) and plasma corticosterone profiles (Fig. 7b) did not differ between WT and PKR−/− mice during the course of infection. Hence, the altered behavioral components in PKR−/− mice also did not result from an impaired host defense to the *E. coli* infection or from a dysregulated corticosterone response. However, PKR−/− mice displayed a delayed induction of CRH after *E. coli* challenge (Fig. 7), suggesting a postponed CRH response in these mice that may possibly modulate sickness.

As expected, genetic deletion of PKR suppressed peripheral inflammatory responses to *E. coli* infection. Previous studies have demonstrated that deficiency of PKR attenuates plasma IL-6 and IL-12 increases after systemic LPS challenge [48] and that PKR regulates inflammasome activation [51] and TLR2/TLR4-dependent cytokine release [46] from cultured macrophages. Moreover, PKR shows crosstalk with multiple inflammatory pathways including NF-κB [47, 49], MAPK [48], IRF-1 [49], and JNK signaling cascades [46, 47]. Our findings are therefore in agreement with these earlier reports. Since systemic inflammatory mediators can communicate with the brain by neural and humoral routes to trigger neuroimmune activation [3, 19], we asked whether PKR deficiency could also suppress neuroimmune responses after *E. coli* challenge.

To our surprise, WT and PKR−/− mice displayed mostly similar neuroimmune changes. This is in contrast to several earlier studies, which showed that pharmacological inhibitors of PKR can abolish inflammatory responses in glial cultures [50, 52, 80]. Until recently, it has also been demonstrated that genetic deletion or pharmacological inhibition of PKR reduces neuroinflammation in animal models of viral encephalomyelitis [53] and excitotoxic injury [54]. It should be noted that in these studies, inflammatory changes were initiated directly in glial cultures or by direct brain insults, whereas in our scenario neuroimmune activation occurred subsequently to subcutaneous inflammation caused by *E. coli*. This notable deviation suggests that PKR in the brain and PKR at peripheral tissues may act differentially to affect neuroimmune responses under different causes. It is known that systemic immune challenge can promote the recruitment of neutrophils [81–84] and monocytes [85, 86] into the brain and that infiltrating leukocytes can influence inflammatory changes in the brain [84, 87, 88] and even LPS-induced depression-like behavior [84]. For instance, a single injection of LPS dose dependently elevates the number of infiltrating neutrophils into the facial nucleus within 48 h, a phenomenon that can persist up to at least 96 h [83]. Given that *E. coli* challenge led to a sustained infection along with increased inflammatory factors up to 120 h, it is expected to have a similar effect as a high dose of LPS to cause considerable leukocyte infiltration into the brain. One possibility could be that PKR may modulate this leukocyte infiltration process. Should this be the case, even if deficiency of PKR can downregulate the neuroimmune changes mediated by glial cells, this effect may be masked by the inflammatory responses elicited by the infiltrating leukocytes. Indeed, PKR−/− mice display enhanced T cell recruitment into the brain during viral encephalomyelitis [53], suggesting that PKR may also regulate the entry of other leukocyte cell types into the brain. Further investigation along this direction may provide clues as to how PKR controls neuroimmune activation during systemic immune insults.

Irrespective to the cause for the similar neuroimmune responses, PKR−/− mice did show decreased peripheral inflammatory changes. This finding led us to predict that sickness might also be diminished in PKR−/− mice, since sickness can be ameliorated by blockade of peripheral cytokine synthesis or their effects [27, 29, 32, 89], or by genetic deletion of NF-κB [90]. Therefore, we monitored both core (anorexia, motor impairments) and behavioral (burrowing and exploratory deficits, social withdrawal) components of sickness [1, 5, 19, 71, 91] in WT and PKR−/− mice after *E. coli* challenge. Previous literature has shown that these two types of sickness components can be temporally [11, 92–94] and pharmacologically [27, 29, 93, 94] dissociated, indicating that the two types of sickness components involve different regulatory mechanisms. For example, LPS-induced deficit in burrowing can persist up to 24 h, when the decrease in locomotor activity is no longer observed [11]. On the other hand, while administration of cyclooxygenase (COX) inhibitors can ameliorate both core (hypothermia, impaired locomotor activity) and behavioral (decreased burrowing) components of sickness following LPS challenge, blockade of peripheral cytokine synthesis by dexamethasone or dexamethasone-21-phosphate only attenuates LPS-mediated hypothermia [27, 29]. Here, our data demonstrate a rather unexpected finding. Knockout of PKR in mice did not elicit any reduction in sickness responses. Instead, the core components of sickness were similar between both strains of mice, and the behavioral components of sickness were only observable in PKR−/− mice. Of particular interest is that PKR−/− mice showed decreased activities in the open field test, object investigation test, and social interaction test even at 120 h, although these changes were absent in WT mice at the same time point. It should be noted that these exploratory and social deficits observed only in PKR−/− mice were not simply because of a general decrease in motor activity, because rotarod performance had been restored to the control level by 96 h. Thus, this is an indication that deficiency of PKR primarily affected the behavioral components of sickness but not the core components. We did not perform these tasks at an earlier time because we had specifically wanted to distinguish the roles of PKR on the core and behavioral components of sickness. Moreover, both strains of mice displayed decreased rotarod performance at 24 h, indicating that the use of these behavioral assays at or before this time would not allow us to separate the effects of the core and behavioral components of sickness. Given that LPS is well reported to abolish exploratory activity [10, 95] and induce social withdrawal [96–98] in WT animals within several hours after systemic administration, it is likely that *E. coli* can also lead to these deficits in the two strains of mice under this time frame.

Interestingly, we did not detect any change in the burrowing activity of WT mice after *E. coli*. This would seem contradictory to earlier reports which demonstrated that LPS acutely decreases burrowing in WT animals [11, 27, 28]. However, it should be emphasized that these studies have used food pellets as the burrowing substrate, whereas here we used bedding material (wood chips) to avoid interference effects on the measurements of food consumption. It has been documented that different types of burrowing substrates can greatly influence the burrowing activity measured in rodents [99, 100]. C57BL/6 mice typically burrow a greater proportion of the burrowing substrate when the burrowing tube is filled with bedding material than when it is filled with food pellets. For example, Kir6.2 knockout mice display severely impaired burrowing as compared to WT mice when food pellets are used, but this difference becomes less obvious when the burrowing substrate is replaced with bedding material [99]. Based on these studies, it could be deduced that bedding material is more preferred than food pellets as a burrowing substrate by C57BL/6 mice, such that it will be harder to detect phenotypic differences with bedding material than with food pellets. Perhaps WT mice would have displayed burrowing deficits after *E. coli* challenge if the burrowing tubes had been filled with food pellets.

Next, we asked whether the alterations in the behavioral components of sickness of PKR−/− mice would be correlated with exaggerated bacterial load. It has been well reported that loss of PKR can often enhance viral replication [41, 44, 101, 102]. Another study has shown that PKR is involved in resistance to *Toxoplasma gondii* [45]. To our knowledge, however, the effect of PKR in bacterial infection has been little investigated [51]. Here, we made use of two different methods, i.e., microbiological plate count and quantification of 16S rDNA by real-time PCR, and show that bacterial titers were similar between the two strains of mice throughout the course of infection. These results imply that altered sickness in PKR−/− mice was not due to an impaired host defense to *E. coli*. In fact, even if PKR deficiency does affect *E. coli* infection, it would likely suppress it. This point is supported by another study by Lu et al., in which PKR−/− mice had reduced bacterial titers following *E. coli*-induced peritonitis [51].

We then investigated if PKR could modulate the CRH response to *E. coli* challenge. Previous studies have indicated that systemic inflammation leads to CRH induction and release from paraventricular nucleus (PVN) neurons of the hypothalamus [56, 77]. Furthermore, administration of CRH into rodents acutely reduces exploratory activities to novel environments [103] and to novel individuals [104]. These behaviors are quite similar to what was observed in PKR−/− mice at 120 h after *E. coli* challenge. Our data indicate that while *E. coli* upregulated CRH expression at 4 h in the hypothalamus of WT mice, such increase was not found in PKR−/− mice. This finding is not surprising, given that systemic inflammation induces CRH, and PKR−/− mice showed reduced peripheral inflammation. However, at 48 h after *E. coli* infection, there was a significant elevation of CRH in PKR−/− mice but not in WT mice. Hence, it appears that PKR deficiency can delay CRH induction in response to *E. coli*. It is also possible that knockout of PKR may extend the CRH response period, although this requires validation in a more detailed temporal manner. The altered CRH response in PKR−/− mice suggest that these CRH-mediated effects may also be postponed or extended, which can possibly alter the behavioral components of sickness in PKR−/− mice. Indeed, chronic administration of CRH into the brain can delay behavioral inhibition induced by LPS [105], thus providing indirect evidence to support this possibility. Future studies should address the role of PKR on the CRH response along with its implication on sickness.

We have known that CRH participates in the HPA axis to stimulate adrenocorticotrophic hormone (ACTH) synthesis and release from the anterior pituitary [77]. ACTH in turn acts on the adrenal cortex, inducing corticosterone production and secretion. Upon systemic LPS challenge, corticosterone is increased in the circulation, and several reports have also demonstrated that endogenous corticosterone can suppress peripheral inflammation and sickness [55, 59, 60]. As PKR−/− mice exhibited a delayed CRH induction, we questioned whether this would result in differential responses of corticosterone to *E. coli*. Surprisingly, there was no significant change in the levels of plasma corticosterone between WT and PKR−/− mice. Plasma corticosterone levels were elevated by *E. coli* to the same extent in WT and PKR−/− mice at 4 h, and by 48 h these increases had disappeared in the *E. coli*-challenged groups. These results suggest that the altered sickness behaviors in PKR−/− mice were not due to a dysregulated corticosterone response. Furthermore, corticosterone profiles in the two strains of mice did not follow the same trend as that of hypothalamic CRH. While CRH induction was blunted at 4 h in PKR−/− mice relative to WT mice, corticosterone was similarly increased in both genotypes. At 48 h, CRH was induced in PKR−/− mice, but plasma corticosterone was not upregulated. Such a discrepancy between the CRH and corticosterone profiles might be due to the following possibilities. Firstly, CRH production is not limited to the hypothalamus. Extra-hypothalamic sources of CRH [106–109] may likely serve as alternative source(s) of CRH in PKR−/− mice, such that hypothalamic CRH production would not be required at 4 h in these mice. Of particular relevance is that CRH is highly expressed at peripheral inflammatory tissues to regulate local immune responses [109]. If deficiency of PKR can hyper-induce CRH at inflammatory sites during the *E. coli* infection, such CRH may potentially spill over into the bloodstream and upregulate corticosterone. Secondly, while corticosterone production is regulated by CRH, it can also be triggered by vasopressin through the type 1b vasopressin receptor in the anterior pituitary [110]. Perhaps PKR can act at the level of vasopressin system, thereby exerting its control over the level of corticosterone. Thirdly, elevated corticosterone has been shown to decrease CRH-R1 mRNA and CRH binding [111]. Since corticosterone was increased before 48 h in PKR−/− mice, this could explain why these mice were unable to mount another wave of corticosterone response even though CRH was induced at 48 h.

It is noteworthy that despite the dissociation between hypothalamic CRH and circulating corticosterone profiles in PKR−/− mice, the possible involvement of the delayed CRH response in altering sickness behaviors should not be simply ruled out. CRH participates in the HPA axis, but CRH effects are not entirely mediated by ACTH or corticosterone. CRH is a neurotransmitter and it can bind to widely distributed receptors CRH-R1 and CRH-R2 in the brain [112]. Indeed, many of the brain regions that express CRH receptors, including the hippocampus, amygdala, hypothalamus, midbrain, and cerebral cortex, are not responsible for ACTH production. Instead, direct manipulation of CRH signaling in these brain structures often results in behavioral changes that are also observed in sickness. For instance, specific deletion of CRH-R1 in midbrain dopaminergic neurons causes anxiety and inhibits dopamine release in the prefrontal cortex [113]. In the same study, it was shown that deletion of CRH-R1 in forebrain glutamatergic neurons decreases anxiety and neurotransmission in the hippocampus and amygdala. Moreover, the direct infusion of CRH into the hippocampus enhances long-term potentiation and improves context-dependent fear conditioning [114]. Interestingly, many of the brain structures that express CRH receptors are also involved in sickness [20], thus supporting for a role of CRH in modulating sickness. A delay in the CRH response in PKR−/− mice suggests that endogenous CRH signaling at an early time after the *E. coli* infection is required for normal sickness development.

In addition to the delayed CRH response, other parameters can be studied in future to better understand sickness in PKR−/− mice. For example, many inflammatory mediators such as NF-κB [90], microsomal PGE synthase-1 (mPGES-1) [115, 116], and transcription factor nuclear factor interleukin 6 (NF-IL-6) [117] have been demonstrated to control sickness and are potential candidates to alter sickness behaviors in PKR−/− mice. In particular, PKR modulates NF-κB activation [47, 49], and genetic deletion of NF-κB abolishes sickness induced by LPS and unmethylated cytosine-phosphate-guanosine motifs (CpG-DNA) [90]. We have focused on quantifying inflammatory gene expression in the brain. This was because inflammatory gene expression serves as a good indicator of local neuroimmune activation and that it could enable us to assess multiple inflammatory markers with a limited amount of sample material. Subsequent investigation of neuroimmune markers at protein level would be important to further characterize neuroimmune responses and their relationships to sickness. For example, iNOS mRNA expression was increased to a slightly lesser degree in the PKR−/− *E. coli* group than in the WT *E. coli* group at 48 h in the hypothalamus. It is known that systemic LPS upregulates iNOS mRNA expression in the hypothalamus [118] and that pharmacological inhibition of iNOS attenuates LPS-induced sickness responses [34]. Our mRNA results raise questions as to whether *E. coli* could indeed upregulate iNOS protein in the hypothalamus and if PKR could modulate iNOS protein production that can potentially affect sickness. Finally, systemic inflammation can activate the indoleamine-2,3-dioxygenase (IDO) pathway, leading to the catabolism of tryptophan (TRP) to kynurenine (KYN) in the brain and blood [94]. In the same study, pharmacological inhibition of the IDO pathway was shown to alleviate LPS-induced depressive-like behavior. In another study, genetic deletion or pharmacological inhibition of IDO ameliorates depressive-like behaviors triggered by an intracerebroventricular injection of LPS [119]. Since PKR−/− mice developed exploratory activity and social interaction deficits up to 120 h, a dysregulation of the IDO pathway is another reasonable possibility that may account for these altered sickness behaviors.

A major limitation of our study is that we have used a general knockout approach to model the effect of PKR on neuroimmune activation and sickness. Since PKR is ubiquitously expressed, it is difficult to pinpoint tissue-specific effects of PKR during systemic inflammation. For instance, we discussed the possibility that PKR deletion at peripheral tissues may enhance leukocyte recruitment into the brain, thereby masking the reduction of neuroimmune responses in glial cells. This issue can be more easily studied if PKR conditional knockout mice were used. Perhaps, it will be beneficial to generate different lines of mice having specific knockout of PKR in target tissues, so as to better characterize the role of PKR during systemic inflammation.

We believe that our findings have important implications. Firstly, we identify a novel role of PKR in regulating sickness, particularly the behavioral components of sickness. As an over-exaggeration of sickness may precipitate depression [5, 19] and/or delirium [3, 6], gaining a better understanding on sickness regulation can shed light on how to fine tune sickness responses. Ideally, one would want to preserve the physiological functions of sickness without causing severe side effects. Secondly, reports in the recent decade have demonstrated that PKR inhibition can be neuroprotective [119–122] and improve cognition [123]. Here, we provide additional information on PKR deficiency during systemic inflammation, and this can complement the earlier studies. While we did show that loss of PKR altered the behavioral components of sickness, we should not simply neglect the desirable effects of PKR inhibition in neurodegenerative processes and memory. Instead, the efficacy of PKR pharmacological inhibitors should be justified after considering both positive and negative effects and taking into account of the patient’s disease status.

## Conclusions

Systemic inflammation leads to neuroimmune activation and sickness. While these phenomena have been well studied, the mechanisms involved in their regulation remain unclear. Our study indicates that genetic deletion of PKR led to alteration in the behavioral components of sickness and this was unlikely to be caused by exaggerated neuroimmune responses, increased bacterial load, or a dysregulated corticosterone response. Instead, PKR deficiency delayed CRH induction after immune challenge by *E. coli*. Future investigations can address the role of PKR in the CRH response, together with its relation to sickness.
